# On the Premature Dementia of Puberty and Adolescence

**Published:** 1903-05-23

**Authors:** W. Lloyd Andriezen

**Affiliations:** late Deputy Medical Superintendent Darenth Asylum; formerly Medical Officer and Pathologist at the West Riding Asylum


### Hospital Clinics and Medical Progress.

### ON THE PREMATURE DEMENTIA OF PUBERTY AND ADOLESCENCE.

By W. Lloyd Andriezen, M.D.Lond., late Deputy Medical Superintendent Darenth Asylum ; formerly Medical Officer and Pathologist at the West Riding Asylum.

The period of puberty and adolescence is one of the most important, the most critical, and psychologically the most interesting in human life. Although mental disorders of various forms have long been known to occur in this period of life it is only during recent years that we have come to recognise the existence of a special group of mental disorders peculiar to this period of development, marked by special features as regards onset and early symptoms, running a characteristic course and tending to terminate in early dementia. To this group of mental affections the term *dementia præcox*, or the precocious dementia of adolescents, is applied, while it is also spoken of as "adolescent insanity" by some authors.

Pinel and Esquirol, the founders of Modern Psychiatry, writing a century ago, regarded "adolescent insanity" as a form of idiocy. Modern conceptions of idiocy do not, however, permit us to place adolescent insanity under that heading. Morel, of Belgium, in the middle of the nineteenth century, was the first to recognise clearly that adolescent insanity was an early form of dementia (*démence précoce*), and to regard its occurrence as a "stigma of mental degeneration." At a later period Hecker (1870) and Kahlbaum (1874) were able to show that two main varieties of adolescent insanity could be distinguished, and to these varieties the terms hebephrenia and catatonia were applied. Recent observations made by alienists in Germany, France, the United Kingdom and America have tended largely to concur in including under the term dementia præcox a large and distinctive group of the insanities of puberty and adolescence. The term dementia præcox was first proposed by Pick, of Prague, in 1891, to embrace and include all "cases of mental degeneracy of hereditary origin stranded on the rock of puberty"—a picturesque descriptive phrase which we owe to Schüle. The term dementia præcox as used in this article comprises a distinctive group of the insanities of puberty and adolescence, the subjects of which, in addition to other features, show a tendency to pass into early and premature dementia.

*Stages of Evolution of Dementia Præcox.*—The stages of evolution of dementia præcox are briefly as follows:—First, there appears a state of mental dulness (loss of the mental brightness of later childhood and youth) with apathy (incapacity for attention) and abulia (weakening of the power of will and of application). The moral disposition is also blunted and there is made manifest a lack of affection for relatives and friends. These symptoms in their entirety constitute the central strand or core of dementia præcox. Many persons who have attained to the age of puberty may develop this degree of mental degeneration but progress no further into dementia. Such persons may remain constitutionally in this state for life—failures to attain the normal level of intellectual and moral development. They are not always or even often found in asylums for the insane. Many vagrants, tramps, petty criminals and prostitutes belong to this class or group of degenerate humanity in which failure of higher development attests itself at the critical period of puberty and adolescence.

The second stage of dementia præcox implies a more serious and profound mental affection. It consists of states of mental confusion, hallucinatory excitement, and delusions supervening on the basis of the mental failure or defect referred to in the foregoing paragraph as the "first stage." In this, the second stage, the patient seems to be wrapt up in a waking delirium or state of dream-like mental confusion—a condition presenting some analogy to the milder forms of alcoholic delirium tremens. Patients in this stage of dementia præcox are obviously insane. Mobile and fleeting hallucinations of hearing, sight, smell, and the other senses, and delusions of persecution by malign and unseen agencies constitute the contents of this delirium or state of mental confusion. The hallucinations are annoying and offensive, male patients have delusions of poisoning and of persecution, female patients of being pursued, maltreated or ravished. Such states of delirium or mental confusion may persist long (for weeks and months) or may occur in short sharp attacks (lasting a few hours or days) with intermissions of irregular character. Their pathogenesis is ho[illegible]ver probably the same, they are the resultant of some unknown toxæmic condition operating on a brain which is hereditarily unstable and defective. So-called melancholia proper, a disease of later life, is rare in puberty and adolescence, and in its classical form is scarcely ever met with at this period of life. This second stage of dementia præcox is accompanied by vasomotor disturbances, cold and clammy extremities which tend to cyanosis, anorexia, some loss of weight, a furred tongue, dilated sluggish pupils, slight elevations of temperature (one-half to one degree Fahr.), constipation, cessation of the menses in the female, exaggeration of the knee-jerks and tendon-reflexes, increased irritability of the muscles to tapping and an undue facility of the skin and subcutaneous tissues to bruising.

The third stage is one of easily recognisable dementia and is a sequel to the preceding stages. This stage is characterised by fixed delusions of grandiose, persecutory, or hypochondriacal character, stereotyped attitudes and acts, dirty and degraded habits, echolalia, and general deterioration of mind and body. As the dementia becomes more marked the delusions grow less potent and gradually disappear, though traces of them may remain and manifest themselves from time to time. The terminal dementia is either of the apathetic, vegetative and torpid type, or assumes a restless, bizarre, automatic repetitive form, characterised by purposeless movements, gestures, mutterings, and stereotyped attitudes and acts.

In the milder forms of dementia præcox the patient may, after an attack of mental disorder in puberty or adolescence, exhibit only a moderate degree of permanent mental enfeeblement which need not incapacitate him for the simpler duties of social life. This occurs in about 10 per cent. of cases of dementia præcox. Such cases, however, always constitute an element of danger or trouble to society when at large. Many tramps and vagrants, petty criminals and prostitutes belong to this category of mental degeneration, but as they seldom find their way into asylums for the insane they do not usually come under the cognisance of lunacy law. Hysterical and hebephrenic cases (a description of which is given below), are often met with in this category of degenerates.

To sum up, therefore, we may state that dementia præcox is not a motley group of varied and unrelated vesanic insanities, but is a special distinctive class of the insanities characterised by mental enfeeblement supervening in early life (puberty and adolescence), slowly progressive in character, and marked in its early stages by attacks of mental confusion, polymorphic hallucinations and delusions of a fleeting unstable and unsystematised nature, and terminating in moderate or marked permanent dementia.

*The Psychogenesis of Adolescence and its Relation to Mental Disorder.*—The rapid mental and bodily changes occurring during the period of puberty and adolescence constitute a critical state so far as it concerns the mental and moral equilibrium of the individual. The pubescent boy or girl becomes more and more subjected to social restraint. Parents impose laws and conventions of which the child had hitherto remained ignorant. A new world of ideas and aspirations dawns upon the youth and maiden. Meanwhile the sexual organs (ovaries, testes, external genitals, and in the female the mammæ) develop rapidly, the larynx in both sexes and the pelvis in the female undergo enlargement, and increase of stature and of strength are attained. The sportive instincts and the exuberance of spirits of childhood are modified and guided into more definite channels. In intelligent children great capacity for reading and absorbing knowledge marks this stage, while memory is both facile and powerful. An undue estimate of individual capacity is engendered in the subject of these budding powers; the youth feels he can conquer the world, partly because the correcting and disillusioning touch of time and experience has not as yet been felt, and partly because the vigorous metabolic and nutritive processes of growth at this period beget within the organism feelings of personal strength and power which, again, experience can only correct. Hence an undue conceit of self, and an uncritical self-assurance and aggressiveness are common traits of the adolescent period of life especially in boys. Creative, artistic, poetic, and constructive talents also show themselves in boys, and to a less extent in girls, at this stage. The religious and erotic instincts now begin to unfold themselves with great fervour as shown by religious zeal and "conversions," on the one hand, and by ardent inclinations to romance, love and heroism on the other. The blind organic instincts of sexual life assert themselves at first in the form of vague longings and aspirations to love, romance, and self-sacrifice. These growing traits and tendencies may develop normally, slightly, or excessively according to hereditary endowment and the action of circumstances, but what characterises them all in common is that there is but little intellectual basis or motive force behind them at first. They have a blind organic non-rational potency of their own which it becomes the office of reason to endow with an intellectual colouring and clothing. But whenever the instinct or organic impulse becomes strong the wisest maxims of intelligence are overcome when the two come in conflict. In psychological language the fact may be restated in the following terms—namely, that the inhibitory powers of the highest cortical centres prove insufficient when they come into conflict with or attempt to inhibit the organic instincts and impulses referred to.

Among the more striking abnormal manifestations of puberty and adolescence, not however amounting to insanity, may be mentioned the tendency to solitude and seclusion shown by some, or the opposite condition of aggressiveness and hooliganism displayed by a few, or to the special development of hypochrondriacal depression, religious exaltation, delusions of sinfulness including "sin against the Holy Ghost," sexual perversion or precocity, excessive cruelty and destructiveness, extraordinary and reckless bravery, and hysterical romancing and lying (pseudologia fantastica) to court attention, manifested by others. These, however, are but minor phenomena; they are, as a rule, transitory manifestations and oscillations of a growing ego whose varied and conflicting intellectual, instinctive, and organic elements are seeking to attain equilibrium and harmony of action. Like the tics and choreiform disturbances of puberty they tend to disappear within a few years. In cases of insanity arising at this stage and on the basis of such developing factors the discordances and abnormalities above referred to are displayed more strikingly. The intelligence is markedly obscured, the power of attention and of volition are weakened or paralysed, while a grotesque combination and over-action of the organic instincts and impulses proper to this period of life are manifested. The symptom-complex includes such phenomena as apathy, stubborn silence and mutism, an apparent arrest of the capacity for attention almost amounting to stupor, a shallow melancholy varied by outbreaks of liveliness and quixotic demonstrations, the exhibition of histrionic attitudes and silent religious postures varied occasionally with shouting and silly chatter, and a stupid passive or sub-active resistance to everything required to be done by the patient (negativism). Rapid and fickle changes of mood are observed in many patients of this class. Delusions of a persecutory, erotic, or mystico-religious type are very prone to be developed, and occasionally the patient may, without warning, give way to violent and dangerous impulses lasting for a few minutes or hours. Girls are exposed to greater risk of mental disorder than boys during the period of puberty—the period from 15 to 18 years of age —but the period of adolescence—18 to 25 years of age—is fraught with greater risk of mental disorder to the male. On the whole, dementia præcox occurs about twice as frequently in the male as in the female.

*(To be continued.)*

